# Supplementary figures and images for: A novel deep learning-based point-of-care diagnostic method for detecting Plasmodium falciparum with fluorescence digital microscopy
Source: PLoS One. 2020 Nov 17;15(11):e0242355. doi: 10.1371/journal.pone.0242355 (PMC7671488; doi:10.1371/journal.pone.0242355)

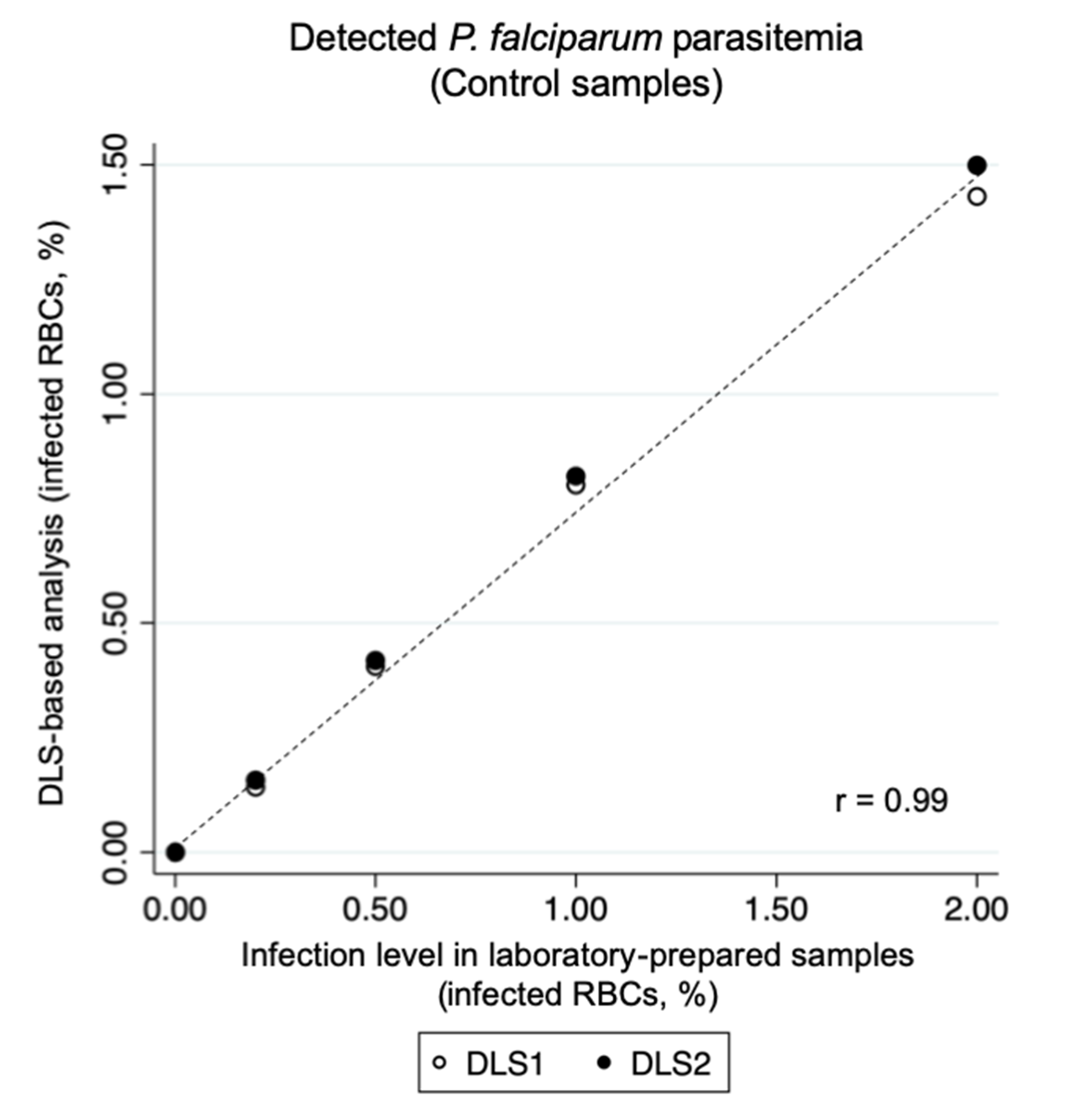

Supplement: S1 Fig — Results from deep learning-based analysis of control samples, prepared from blood cultures in laboratory-conditions with known levels of P. falciparum infections (0% and approximately 0.2%, 0.5%, 1% and 2% levels of parasitemia, respectively). Correlation between results measured with the Pearson's product-moment correlation coefficient and showing an almost perfect level of correlation (r(7) = 0.99). (TIF) [file pone.0242355.s001.tif]

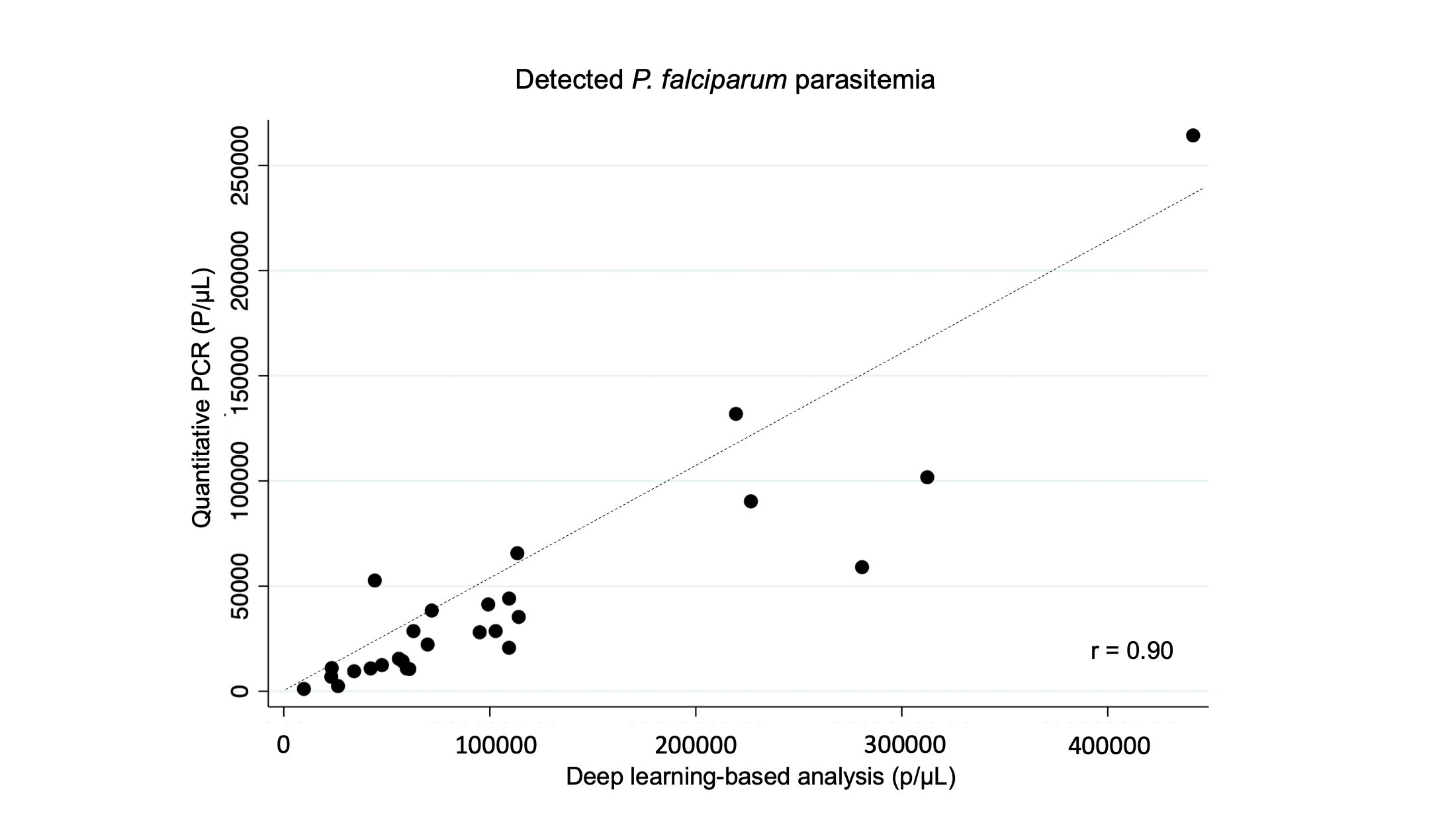

Supplement: S2 Fig — Detected level of malaria infection before initiation of treatment, as determined by analysis with the deep learning-systems (DLSs), compared to quantitative PCR-based analysis of samples from a subset of patients. DLS-detected parasitemia calculated based on an assumed amount of 5,000,000 RBCs per μL of blood. Correlation between results measured with the Pearson's product-moment correlation coefficient and showing a high level of correlation (r(27) = 0.90). (TIF) [file pone.0242355.s002.tif]

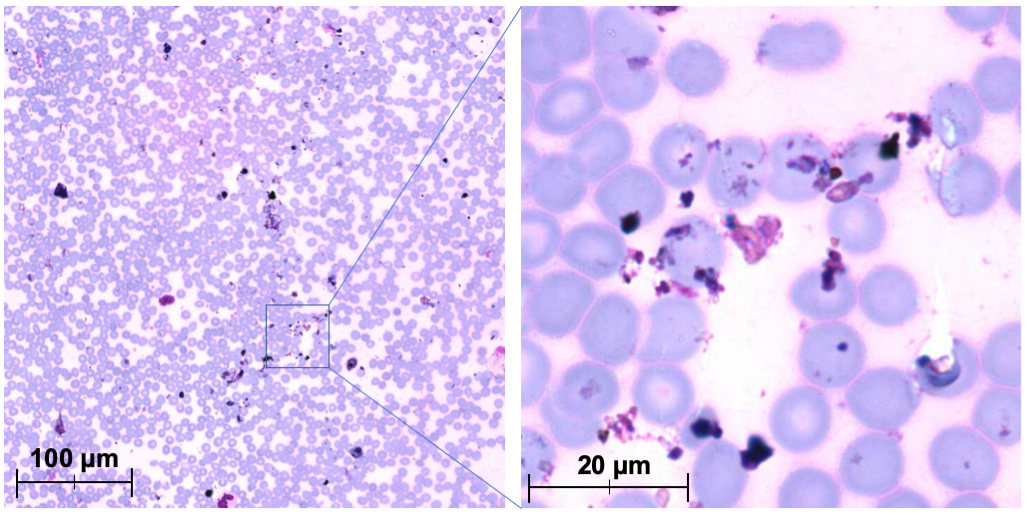

Supplement: S3 Fig — Digitized Giemsa-stained thin blood smear from patient in study cohort. Images showing sample with high amounts of visible artefacts and debris. (TIF) [file pone.0242355.s003.tif]

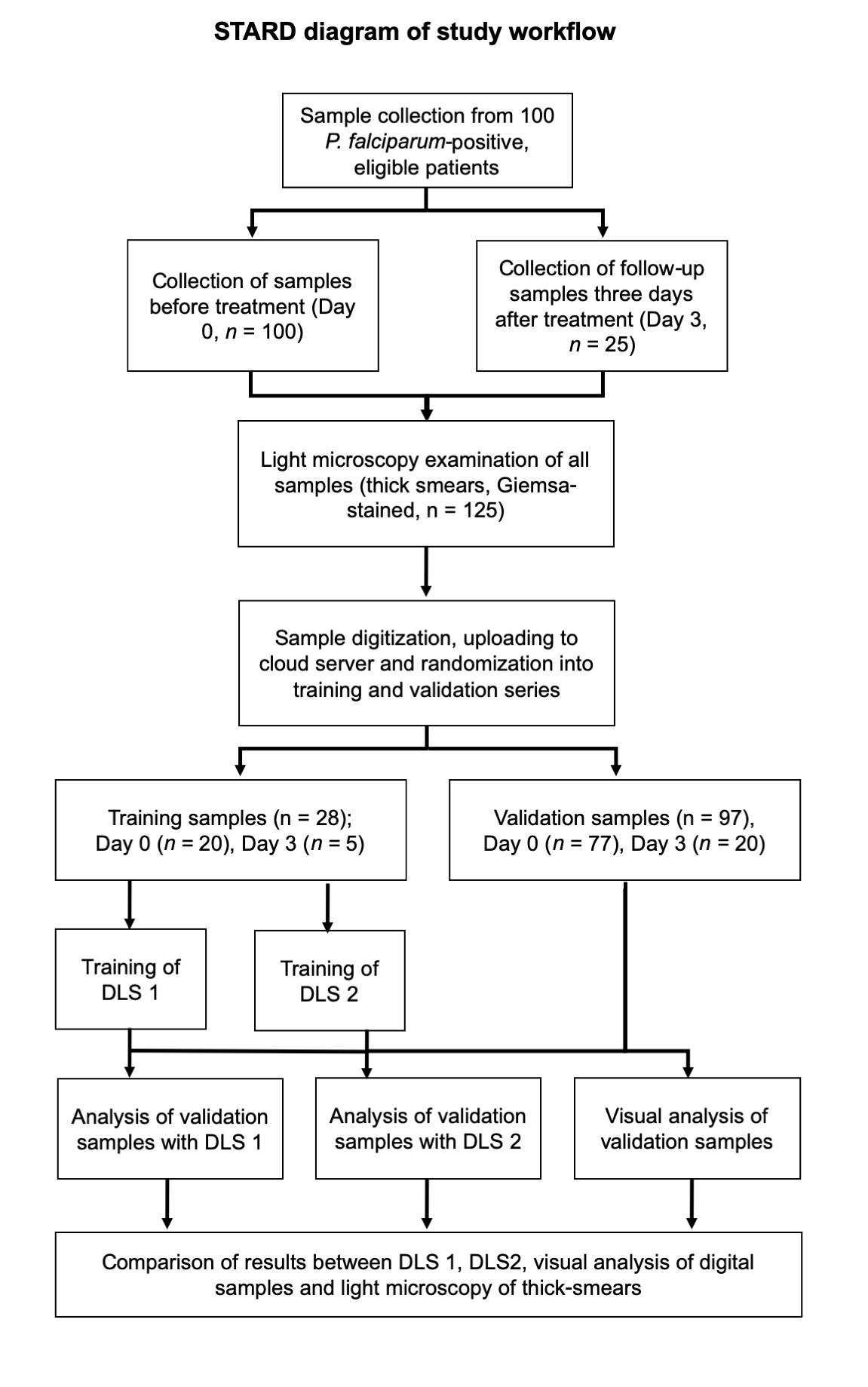

Supplement: S4 Fig — STARD diagram of study workflow and sample processing. (TIF) [file pone.0242355.s004.tif]
